# A Near-Zero Refractive Index Meta-Surface Structure for Antenna Performance Improvement

**DOI:** 10.3390/ma6115058

**Published:** 2013-11-06

**Authors:** Mohammad Habib Ullah, Mohammad Tariqul Islam, Mohammad Rashed Iqbal Faruque

**Affiliations:** 1 Institute of Space Science (ANGKASA), National University Malaysia, Bangi, Selangor 43600, Malaysia; E-Mails: habib_ctg@yahoo.com (M.H.U.); rashedgen@yahoo.com (M.R.I.F.); 2 Department of Electrical, Electronic and System Engineering, Faculty of Engineering and Built Environment, National University Malaysia, Bangi, Selangor 43600, Malaysia

**Keywords:** near zero refractive index (NZRI), meta-surface structure (MSS), square loop, multi band antenna, performance enhancement

## Abstract

A new meta-surface structure (MSS) with a near-zero refractive index (NZRI) is proposed to enhance the performance of a square loop antenna array. The main challenge to improve the antenna performance is increment of the overall antenna volume that is mitigated by assimilating the planar NZRI MSS at the back of the antenna structure. The proposed NZRI MSS-loaded CPW-fed (Co-Planar Waveguide) four-element array antenna is designed on ceramic-bioplastic-ceramic sandwich substrate using high-frequency structure simulator (HFSS), a finite-element-method-based simulation tool. The gain and directivity of the antenna are significantly enhanced by incorporating the NZRI MSS with a 7 × 6 set of elements at the back of the antenna structure. Measurement results show that the maximum gains of the antenna increased from 6.21 dBi to 8.25 dBi, from 6.52 dBi to 9.05 dBi and from 10.54 dBi to 12.15 dBi in the first, second and third bands, respectively. The effect of the slot configuration in the ground plane on the reflection coefficient of the antenna was analyzed and optimized. The overall performance makes the proposed antenna appropriate for UHFFM (Ultra High Frequency Frequency Modulation) telemetry-based space applications as well as mobile satellite, microwave radiometry and radio astronomy applications.

## 1. Introduction

In recent years, engineered materials have been used in antenna technology by several researchers. After a pioneering investigation of numerous properties of artificial materials with negative permittivity and permeability, substantial research attention has been turned to the use of meta-surfaces [1,2]. One of the potential applications of meta-materials or meta-surfaces is enhancement of antenna performance [3,4]. Micro-strip patch antennas have become quite popular due such attractive features as low cost, light weight, low profile, design simplicity and easy manufacturability [5]. However, these low-profile patch antennas suffer from low gain and directivity, which limit the range of applications. High gain and directivity are required for these antennas to be suitable for long distance applications. As a result, several researchers have proposed numerous techniques to improve the gain and directivity of these antennas in recent decades. 

One of the most common techniques used to improve antenna gain and directivity is the use of multiple patch configurations, but this approach can result in an unstable radiation pattern with unexpected ripples [6]. Use of stacked/multilayer patches and/or air gaps between patches was proposed to enhance the gain [7]; however, this method increases the overall antenna volume. Frequency selective surface (FSS) and/or electromagnetic bandgap (EBG) structures were also proposed by several researchers for antenna gain improvement [8,9]. Nevertheless, these techniques increase the overall thickness of the antenna, or the enhancement of the antenna performance is insufficiently improved and further improvement is still necessary. Partial reflective surfaces (PRS) linked with an artificial magnetic conductor (AMC) were proposed to overcome the thickness problem as well [10,11]. The total thickness is decreased using this approach, but the aperture efficiency remains lower. A straightforward method for improving the patch antenna bandwidth and gain is to use a thicker substrate that increases the fraction of total power excitation towards the surface wave. The increased power of the direct radiation causes degradation of the radiation pattern and efficiency of the antenna.

Artificial materials have been used in different forms to improve the antenna gain and directivity [2,4,12]. A revolutionary study was performed on the use of left-handed materials (LHM) to improve antenna directivity in which the Snell-Decartes laws were applied with an near-zero refractive index (NZRI) over the superstrate [7]. Two typical types of directive antennas are parabolic antennas and very large array antennas. The bulk and curved surface of parabolic antennas limit their use in many commercial applications. Additionally, the complex feeding mechanism and losses in the feeding network are two major disadvantages associated with large micro-strip array antennas [13]. One of most effective solutions to these problems is the use of a planar meta-surface embedded with the patch antenna [12]. Many researchers have engineered NZRI materials for different applications [14,15]. One of the most attractive properties of NZRI materials is that the electromagnetic field inside a zero refractive index medium propagates with a near-zero phase variety. Among the possible applications are microwave to optical frequencies for use in such as controllable “smart” surfaces, miniaturized cavity resonators, novel wave-guiding structures, angular-independent surfaces, and absorbers, among others. Meta-surfaces could be a potential candidate for substantial antenna performance enhancement. The key features of the proposed NZRI meta-surface structure (MSS) are low profile, ease of integration with active microwave components and coupling reduction between radiating elements in the antenna array [16]. Therefore, no cavity is necessary because the meta-surface is directly illuminated by the antenna element. However, to transform this concept effectively to smart antenna technologies, it is appropriate to assimilate the NZRI MSS with micro-strip antennas.

In this paper, we propose the design and experimental analysis of a NZRI MSS-backed CPW-fed 2 × 2 square loop antenna array for multiband applications. Assimilation of the proposed NZRI MSS at the back of the radiating patch significantly enhances the gain and directivity without increasing the overall size. The radiation performance makes the proposed antenna suitable for UHF FM telemetry-based space applications, mobile satellites, microwave radiometry and radio astronomy applications. The gain and directivity profile meet the basic requirements of the antennas for these long distance applications.

## 2. Design Specifications

The design of the proposed NZRI MSS-backed patch antenna was performed using a widely used finite element method based on a three dimensional (3D) full-wave high-frequency structure simulator (HFSS version 13.0.2). The proposed CPW-fed patch antenna is composed of a 2 × 2 square loop element array on 2 mm thick ceramic-filled bioplastic high-permittivity (*ε*_r_ = 15) dielectric material substrate backed by a 7 × 6 set of NZRI MSS and a slotted ground plane. The design procedure begins with determination of the dimensions of the proposed antenna. The length and width of the proposed antenna are chosen with the aid of a well-established mathematical formulation [17].

**Figure 1:**
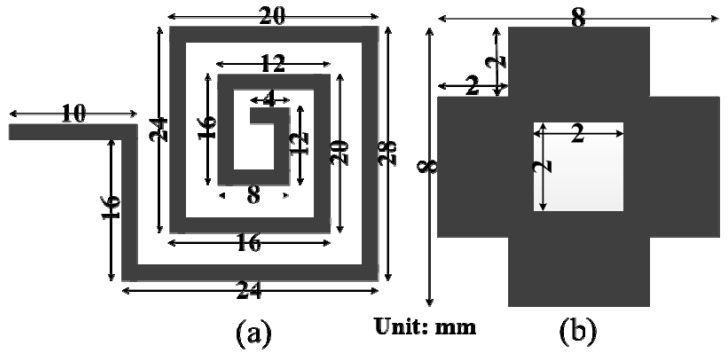
Design schematic of (**a**) the array element; (**b**) the meta-surface structure (MSS) element of the proposed antenna.

**Figure 2:**
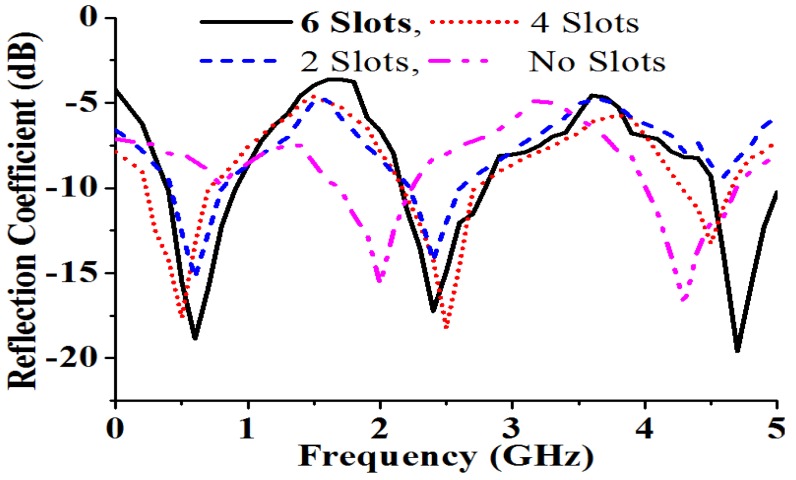
Effect of slot loading in the ground plane of the proposed near-zero refractive index (NZRI) MSS antenna.

**Figure 3:**
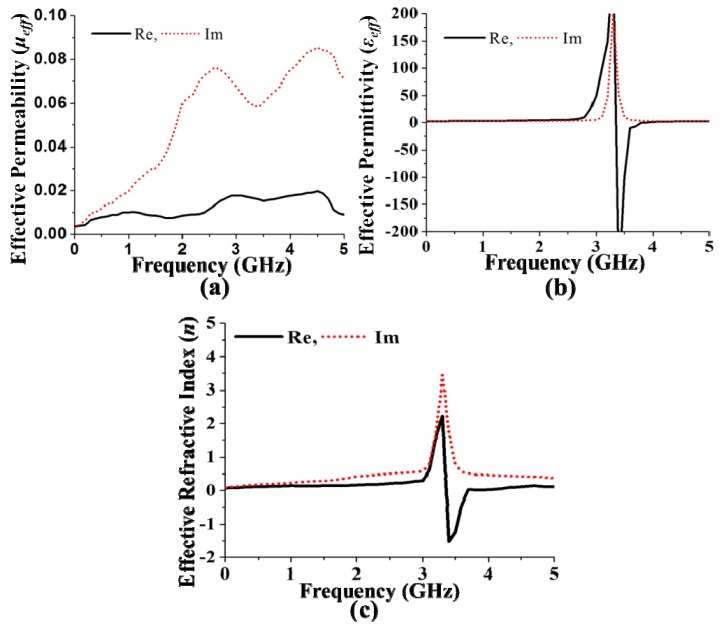
(**a**) Effective permeability; (**b**) effective permittivity; and (**c**) refractive index of the proposed MSS.

**Figure 4:**
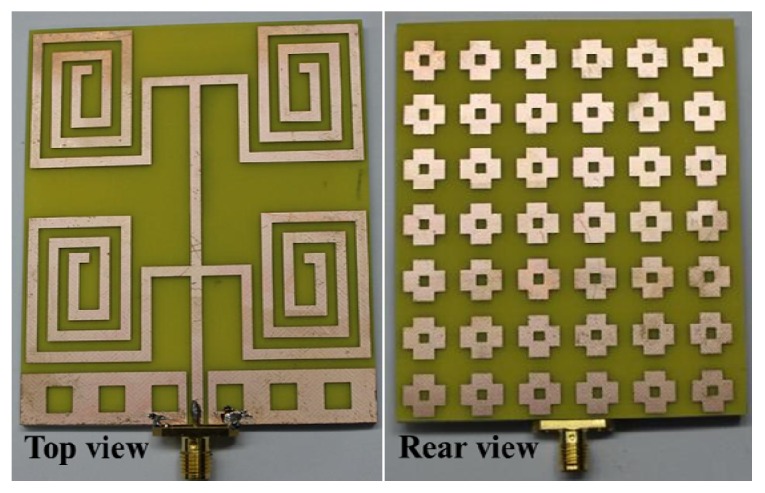
Photograph of the proposed NZRI MSS-backed antenna prototype.

Figure 1 shows the design scheme of the array element and the MSS element of the proposed antenna. The square loop of the array element is constructed with 2 mm thick symmetric lines and four elements connected with a 2 mm wide and 68 mm long micro-strip feed line. A standard 50 Ohm SMA connector is used to connect a coaxial probe at the end of the feed line for excitation. Generally, the impedance bandwidth of the patch antenna degrades with the use of a high permittivity dielectric substrate material. However, this shortcoming has been resolved by introducing square slots in the ground plane. The effect of the slots in the ground plane on the reflection coefficient of the proposed NZRI MSS antenna is shown in Figure 2. In the proposed design of the NZRI MSS, a four-petal shape was chosen, which has been theoretically studied and analyzed using computer aided tools. The symmetrical and uniformly distributed four-petal MSS elements are arranged in a 7 × 6 matrix pattern. The four-petal MSS element is obtained by cutting 2 mm × 2 mm slots from the corners and the center of a rectangle. The shape and distribution of the MSS elements was determined using the finite-element-method-based HFSS optimization tool to achieve the expected permittivity and permeability. The constrained and superlinearly convergent active-set algorithm used the HFSS optimization tool optimetrics to provide a minimized cost function. The proposed MSS can be characterized as a meta-surface by determining the effective parameters and refractive index. The common approach that determines any structure as a meta-surface is a periodic homogenous structure distributed in a uniform pattern with the desired property of a negative or near-zero index. Several techniques are used to retrieve the effective parameters [18,19]. According to Snell’s law of refraction, a NZRI structure causes the electromagnetic waves to emanate in any direction away from the primary source and to reflect nearly parallel to the normal of the surface of this structure. This characteristic offers an outstanding technique for direction control and directivity increment. The effective parameters of the MSS are obtained with the aid of a widely used finite-element-based and self-consistent method. The basic concept of the effective index of refraction was taken from the well-known Snell’s law of refraction for clear understanding. However, the effective refractive index of the proposed NZRI MSS was determined by implementing the widely used mathematical formulation [20] 
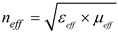
. The effective permeability (μ*_eff_*) and effective permittivity (ε*_eff_*) are shown in Figure 3a,b. The effective epsilon and mu values were extracted with commonly used effective parameter retrieval methods [19,21]. The effective permeability (μ*_eff_*) and effective permittivity (ε*_eff_*) are obtained from the finite-element-method-based simulator HFSS version 13.0.2. It can be clearly observed that the effective permeability is between zero and one in the bands of interest, as expected. With the aid of Snell’s law [22], the refractive index *n* can be defined as that shown in Figure 3c. The refractive index of the proposed MSS is close to zero in the operating bands. Therefore, the effective index of refraction is negative in the 3.4 GHz to 3.6 GHz range. However, the value of the refractive index of the MSS is close to zero and positive within the bands of interest, which are 0.4–0.9 GHz, 2.3–2.75 GHz and 4.6–4.9 GHz. The radiated electromagnetic field of the MSS element produces an internal oscillation, which introduces an identical illumination that improves the overall radiation performance. One of the vital issues in the MSS is interaction with the antenna element. In addition, the MSS reduces the mutual coupling between the antenna array elements. The key approach used in the design is to excite resonant oscillations in the MSS inclusion, and thus the fractional limpidity of the structure can be maintained [23]. The flowing currents induced in the MSS elements at certain frequencies cancel each other, thus permitting the incident wave to circulate. After the successful design and analysis of the proposed NZRI MSS-backed square-loop array antenna, a prototype was fabricated using an LPKF indoor Printed Circuit Board (PCB) prototyping machine. The fabricated prototype of the proposed NZRI MSS-backed antenna is shown in Figure 4.

## 3. Measurement Results

**Figure 5:**
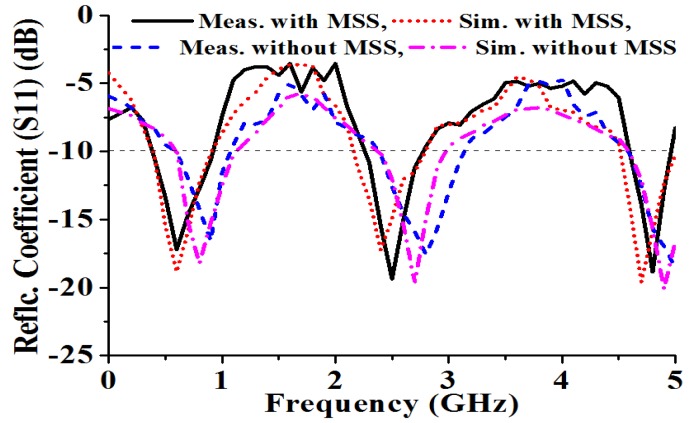
Reflection coefficients of the proposed array with and without the MSS.

The fabricated prototype of the proposed MSS antenna is measured in a standard 5.5 × 5 × 3.5 m^3^ far-field anechoic measurement chamber for experimental verification of the predicted performance parameters. The measured and simulated reflection coefficients of the proposed antenna with and without the NZRI MSS are shown in Figure 5. 

**Figure 6:**
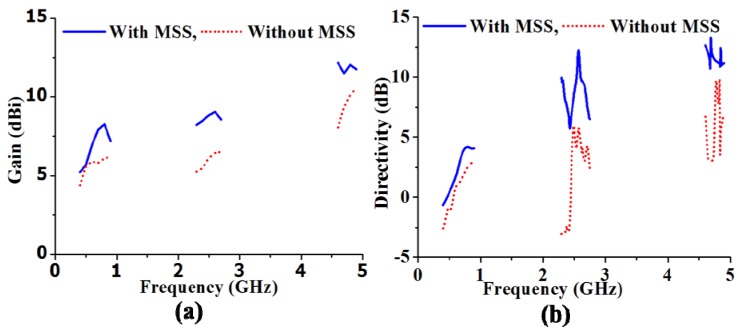
Measured (**a**) gain; and (**b**) directivity of the antenna with and without MSS.

Good agreement is observed between the simulated and measured reflection coefficients of the proposed antenna. The resonances of the antenna without the MSS are slightly shifted toward higher frequencies. However, the proposed and embedded NZRI MSS exhibits the measured bandwidths (S11 < −10 dB) of 500 MHz (0.4–0.9 GHz), 450 MHz (2.3–2.75 GHz) and 300 MHz (4.6–4.9 GHz) centered at 0.6 GHz, 2.5 GHz and 4.8 GHz, respectively. From the reflection coefficient (S11) graph, it can be observed that the proposed antenna could be apposite for UHF FM telemetry-based space applications, mobile satellite, microwave radiometry and radio astronomy applications. The key features of the proposed antenna are the gain and directivity, as illustrated in Figure 6. A standard three-antenna system with two identical horn antennas was used for gain measurement. The gains of the two identical horn antennas are known, and a gain measurement system that follows well-known equations was used for three antennas. From the following equations, the gain of the three antennas (under test) can be calculated because the gains of two horn antennas are known, *R* is the distance between the two antennas and *P_r_* is the radiated power.

Antenna 1 (horn) and Antenna 2 (horn):

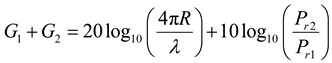
(1)


Antenna 1 (horn) and Antenna 3 (under test):

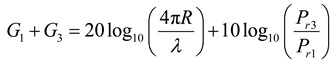
(2)


Antenna 2 (horn) and Antenna 3 (under test):

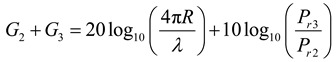
(3)


For directivity *D*, the following equation [17] is used in which U is the radiation intensity and P_rad_ is the total radiated power:

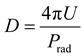
(4)


**Figure 7:**
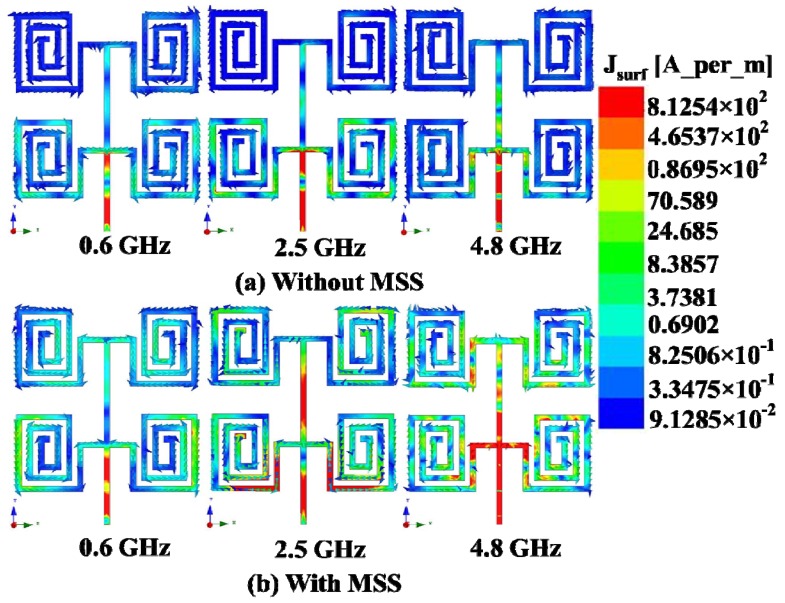
Surface current distribution (**a**) without MSS; and (**b**) with MSS on the radiating patch of the proposed array.

**Figure 8:**
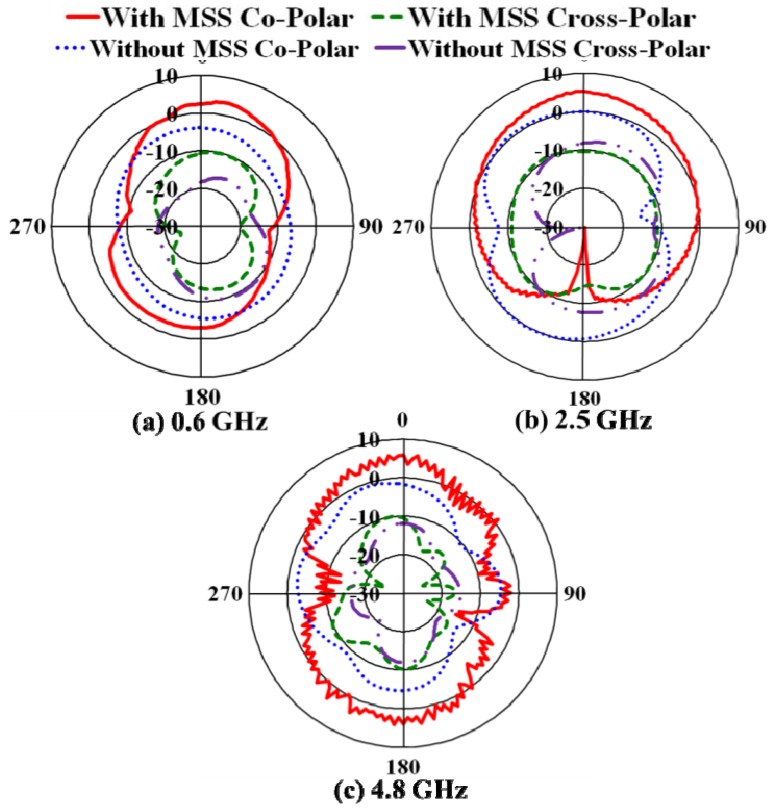
Measured radiation pattern of the proposed NZRI MSS antenna at three resonant frequencies.

It can be clearly observed that the maximum gains are enhanced from 6.21 dBi to 8.25 dBi, from 6.52 dBi to 9.05 dBi and from 10.54 dBi to 12.15 dBi in the first, second and third operating bands, respectively. The directivity of the proposed antenna with the NZRI MSS is considerably increased compared with that of the antenna without MSS. From the directivity result, the NZRI MSS assimilation increases the directivity by 2.99 dB to 4.17 dB, from 5.93 dB to 12.22 dB and from 9.81 dB to 13.23 dB in the first, second and third bands, respectively. The enhanced gain and directivity make the proposed NZRI MSS-embedded antenna with low profile characteristics more desirable than certain existing high-gain antennas [24,25]. For a better understanding of gain enhancement, the surface current distribution over the radiating patch of the proposed antenna with and without MSS combination is presented in Figure 7. It can be observed that the flowing current over the radiating surface is stronger in the higher frequencies compared with that of the lower frequencies. Moreover, it is evident that the intensity of the surface current over the radiating patch of the proposed antenna with NZRI MSS is much higher compared with that of the antenna alone. The measured radiation patterns of the proposed antenna in three resonance frequencies, *i.e*., 0.6 GHz, 2.5 GHz and 4.8 GHz, are shown in Figure 8. In addition to the improvement of gain and directivity, better directional radiation patterns are realized from the MSS embedded antenna compared with those of the antenna without the MSS. It can be observed that the magnitudes of the radiation pattern with the NZRI MSS are higher compared with that of the antenna alone. Moreover, the radiation patterns of the antenna alone are omni-directional, whereas more direct patterns are observed from the NZRI MSS-incorporated antenna. The performance specifications of the proposed antenna with and without the MSS are listed in Table 1. It is clear that the performance of the proposed antenna embedded with NZRI MSS is significantly improved compared with that of the antenna without MSS.

**Table 1:** Performance specifications of the proposed antenna with and without MSS.

Parameter	With MSS			Without MSS		
	1st Band	2nd Band	3rd Band	1st Band	2nd Band	3rd Band
Bandwidth	500 MHz	450 MHz	300 MHz	400 MHz	700 MHz	400 MHz
Maximum Gain	8.25 dBi	9.05 dBi	12.15 dBi	6.21 dBi	6.52 dBi	10.54 dBi
Maximum Directivity	4.17 dB	12.22 dB	13.23 dB	2.99 dB	5.93 dB	9.81 dB

## 4. Conclusions

A new NZRI MSS-embedded CPW-fed antenna is presented in this paper, and the proposed antenna prototype was designed, fabricated and tested. The performance of the proposed antenna is considerably improved by assimilating a 6 × 7 set of four-petal-shaped MSS elements at the back of the antenna structure. A tradeoff between the gain and bandwidth is observed from the experimental results from the proposed antenna with and without the MSS. Furthermore, a more directive radiation pattern was observed from the MSS-based antenna compared with that of the patch antenna alone.
